# 伴PML-RARα融合基因的慢性髓性白血病急性早幼粒细胞白血病变1例报告并文献复习

**DOI:** 10.3760/cma.j.issn.0253-2727.2023.06.014

**Published:** 2023-06

**Authors:** 明锁 刘, 晓雁 韩, 志刚 曲, 秋莲 罗, 康丽 吴, 瑾 陈, 雅君 吴, 婉玲 徐, 席席 杨, 园园 朱

**Affiliations:** 1 义乌市中心医院，义乌 322099 Yiwu Central Hospital, Yiwu 322099, China; 2 浙江大学附属第一医院，杭州 310003 The First Affiliated Hospital, Zhejiang University, Hangzhou 310003, China

在酪氨酸激酶抑制剂（TKI）治疗时代，仍有少数慢性髓性白血病（CML）患者出现急变，预后极差，急变后生存期仅7～11个月[1]。CML急性早幼粒细胞白血病（APL）变罕见，文献仅报道20余例。我们中心报告1例CML确诊20 d后急变为APL的病例并对相关文献进行复习。

## 病例资料

患者，男，27岁。患者入院前因痔疮拟行手术治疗，发现WBC 495.58×10^9^/L，遂至我院血液科就诊。既往史：痛风、痔疮病史数年余，未治疗，否认家族遗传病史。查体：周身皮肤无皮疹、黄染、瘀斑，无贫血貌。双肺呼吸音粗，未闻及干湿性啰音。肝肋缘下未触及，脾甲乙线12 cm。入院后辅助检查，血常规：WBC 449.29×10^9^/L，中性粒细胞占87.3％，淋巴细胞占2.2％，单核细胞占6.3％，嗜碱性粒细胞占3.0％，嗜酸性粒细胞占1.2％，HGB 75 g/L，PLT 287×10^9^/L。肝功能：总蛋白69.6 g/L，白蛋白39.0 g/L，丙氨酸转氨酶3 U/L，天冬氨酸转氨酶15 U/L。肾功能：肌酐318.1 µmol/L，估算肾小球滤过率（eGFR）27 ml·min^−1^·（1.73 m^2^）^−1^，尿酸764.0 µmol/L。凝血功能：凝血酶原时间（PT）17.6 s（参考值9.0～15.0 s），活化部分凝血活酶时间（APTT）36.2 s（参考值21.1～36.5 s），纤维蛋白原1.032 g/L，D-二聚体11 940 µg/L。乳酸脱氢酶850 U/L，免疫球蛋白定量：IgA 2.72 g/L（参考值0.70～4.00 g/L），IgG 15.20 g/L（参考值7.00～16.00 g/L），IgM 1.12 g/L（参考值0.40～2.30 g/L）。尿常规：蛋白质（++），尿隐血（+++）。24 h尿总蛋白601 mg。心脏彩超：左室功能测定正常。胸部CT：肺内未见明显实质性病灶。腹部B超：肝脏形态稍饱满，右肝斜径159 mm，包膜尚光整。脾脏长245 mm，厚64 mm，脾门区可见一大小约37.2 mm×32.3 mm的等回声，界清，CDFI未见明显异常血流信号。双肾大小形态正常，包膜光整。初诊骨髓象：有核细胞量明显增多，粒系增生明显活跃，红系增生减低，占1.5％，巨核细胞382个，产板26个，NAP积分1分。骨髓活检：原始细胞占5％，粒系增生明显活跃，红系增生受抑制，巨核细胞数量大致正常，网硬蛋白轻度增生。骨髓免疫分型：原始髓系细胞群占非红系细胞2.93％，异常粒细胞群占非红系细胞89.52％。综上初步诊断：CML（慢性期）、肾功能不全、凝血功能异常、高尿酸血症、痛风。给予羟基脲1.0 g每日3次口服降细胞治疗，患者白细胞迅速下降，逐渐下调羟基脲剂量至0.5 g每日1次。当WBC下降至39.22×10^9^/L时，停用羟基脲，但WBC仍进行性下降，最低谷时WBC 1.44×10^9^/L，此时骨髓融合基因结果回报：BCR-ABL融合基因26％阳性。骨髓染色体：46,XY,t（9；22）（q34；q11）[10]。综上明确诊断为CML（慢性期）。患者骨髓抑制，予支持治疗。骨髓抑制恢复后WBC进行性升高（30.79×10^9^/L），且合并肾功能不全，予甲磺酸伊马替尼300 mg每日1次治疗，同时外周血可见60％原始细胞，复查骨髓：原始细胞占40.5％，早幼粒细胞占30.5％，考虑CML急变为APL。PML-RARα融合基因51.92％阳性，骨髓染色体：46,XY,t（9;22）（q34;q11.2）[13]/46,idem,der（15）t（15;17）（q24;q21）,der（17）[7]。骨髓荧光原位杂交（FISH）：该细胞分裂象内同时检测到PML-RARα融合基因、BCR-ABL融合基因，证实来源于同一细胞系（图1A、1B）。该患者最终诊断为APL（CML转化）。予全反式维甲酸（ATRA）联合三氧化二砷（ATO）双诱导分化治疗，同时联合TKI（甲磺酸伊马替尼）靶向治疗，治疗第28天复查骨髓示早幼粒细胞占0.5％，疗效评估为完全缓解（CR）。目前患者仍在进一步治疗中。

**图1:**
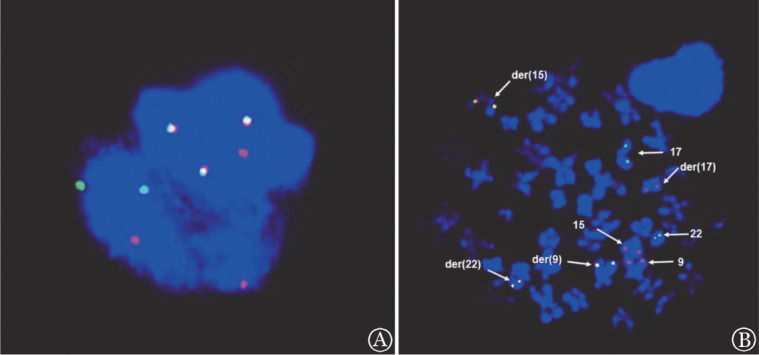
患者荧光原位杂交检测结果 A 细胞间期FISH结果 3F3R2G（F：融合；R：红色；G：绿色）； B 细胞中期FISH结果 3F3R2G。BCR-ABL融合基因为经典易位，产生融合基因BCR-ABL[der（22）]和ABL-BCR[der（9）]；PML-RARα融合基因为非经典型易位，产生融合基因PML-RARα[der（15）]和der（17）

## 讨论及文献复习

TKI的应用显著延长了CML患者的生存期，并将每年CML急变的发生率从20％降低至1％以下[2]–[4]。但仍有少数CML患者会发生急变，急变后预后极差。在TKI治疗时代，急变后CML的生存期通常只有7～11个月；然而在TKI广泛应用前，急变后生存期仅3～4个月[1],[5]。本文中我们报告了1例CML确诊后短期即发生APL急变的罕见病例，并复习了文献报道的22例CML发生APL急变的患者资料（表1）。

**表1:** 22例慢性髓性白血病（CML）急性早幼粒细胞白血病（APL）变患者临床特征

文献来源	性别	年龄（岁）	细胞遗传学	融合基因	CML慢性期治疗	慢性期到APL急变间隔时间	APL急变后治疗	急变后生存期/疗效
Angriman等[6]	女	82	ND	BCR-ABL，PML-RARα	伊马替尼	2年	ND	ND
Matsue等[7]	男	22	46,XY,t(9;22)(q34;q11)[5]/47,idem,+8,t(15;17)(q22;q11-21)[4]	BCR-ABL，PML-RARα	N/A	同时发生	ATRA、化疗、allo-HSCT	移植后118 d
Louwagie等[8]	男	31	46,XY,t(9;22)[40%]/46,XY,t(9;22),t(15;17)(q15;q21)[60%]	ND	阿糖胞苷、6-硫鸟嘌呤	27个月	ND	3个月
Kashimura等[9]	女	69	46,XX,t(9;22)(q34;q11.2),t(15;17)(q22;12)[19]/46,XX[1]	BCR-ABL 88%，PML-RARα 84%	伊马替尼	13个月	ATRA、伊达比星、阿糖胞苷、伊马替尼、ATO	CMR
Laï等[10]	女	85	46,XX,t(9;22),t(15;17)(q22;q21)[35]	ND	ND	10个月	ND	2 d
Colvin等[11]	男	78	46,XY,t(9;22)(q34;q11.2),t(15;17)(q24;q21)[100%]	BCR-ABL 48.25%，PML-RARα 46.44%	伊马替尼、达沙替尼	7年	ATRA、ATO	2个月
Chung等[12]	男	32	46,XY,t(9;22)(q34;q11.2),t(15;?;17)(q22;?;q21)[20]	BCR-ABL，PML-RARα	伊马替尼	6个月	ATRA、伊马替尼	CR
Berger等[13]	男	37	46,XY,t(9;22)[5]/46,XY,t(9;22),t(15;17)[26]	ND	白消安	10个月	柔红霉素、阿糖胞苷	ND
Rosenthal等[14]	女	52	46,XX,t(9;22)(q34;q11.2),t(15;17)(q24;q11.2-12)[9]	ND	羟基脲	3年	米托蒽醌、依托泊苷	6周
Rosenthal等[14]	男	55	46,XY,t(9;22)(q34;q11.2),t(15;17)(q22;q11.2-12)[2]	ND	羟基脲	2年	ATRA、米托蒽醌、阿糖胞苷、依托泊苷	ND
Scolnik等[15]	男	60	46,XY,t(9;22)(q34;q11)[40%]/46,XY,t(9;22) (q34;q11),t(15;17)(q22;q21)[60%]	ND	ND	3年	阿糖胞苷、米托蒽醌、依托泊苷、伊达比星、6-硫鸟嘌呤	3周
Kadam等[16]	男	50	46,XY,t(9;12;22)(q34;q22;q11)[43.8%]/46,XY,t(9;12;22)(q34;q22;q11),t(15;17)(q22;q12-21)[56.2%]	ND	ND	3年	ND	ND
Abe等[17]	男	30	46,XY,t(9;22)(q34;q11)[25%]/46,XY,t(9;22) (q34;q11),t(3;21)(q12;q22)[75%]	ND	白消安	10个月	柔红霉素、6-巯基嘌呤、泼尼松	5个月
Mijović等[18]	女	32	74,,XX,t(9;22),t(9;22)(3n±)	ND	白消安	8年	阿霉素	1个月
Oren等[19]	男	3	亚二倍体t(9;22),iso(17q)^a^	ND	白消安、干扰素、羟基脲、阿糖胞苷	3.5年	化疗	2个月
Misawa等[20]	男	38	46,XY,t(9;22)(q34;q11)[4]/46,XY,t(15;17),t(9;22) [4]/45,X,-Y,t(15;17),t(9;22)[2]	ND	ND	25个月	化疗	1个月
Bobba等[21]	男	26	t(9;22)^a^	ND	N/A	同时发生	阿糖胞苷、柔红霉素、达沙替尼、allo-HSCT	CMR
Cai等[22]	男	31	ND	BCR-ABL 88.36%，PML-RARα 25.4%	羟基脲、伊马替尼	4个月	ATRA、ATO、达沙替尼、伊达比星、阿糖胞苷、allo-HSCT	CMR
Amano等[23]	男	27	51,XY,+der(1)t(1;17)(p11;q11),+7,+8,+8,t(9;22)(q34;q11),22q−	PML-RARα	干扰素	4年	ND	ND
Hogge等[24]	男	38	46,XY,t(9;22)(q34;q11)[4]/46,XY,t(15;17)(q22;q12?),t(9;22) (q34;11)[6]	ND	白消安	25个月	化疗	47 d
Wiernik等[25]	男	48	46,XY[6]/48,XY,+17,+der(1)t(1;?)(p13;?),t(9;22)(q34;q11)[14]	ND	ND	6年	ATRA	>87 d
Wolanin等[26]	男	40	46,XY,t(9;22)(q34;q11.2),t(15;17)(q22;q21)[18]/46,XY[2]	BCR-ABL 41%，PML-RARα 47%	伊马替尼、达沙替尼	17个月	ATRA、ATO、达沙替尼、尼洛替尼	CMR

注 ND：未提供数据；idem：相同染色体；N/A：不适用；ATRA：全反式维甲酸；allo-HSCT：异基因造血干细胞移植；ATO：三氧化二砷；CMR：完全分子生物学缓解；CR：完全缓解。^a^未提供全部染色体信息

由CML急变的APL在分子水平的表现类似于初发APL，需要通过细胞遗传学、荧光原位杂交、分子生物学等方法检测PML-RARα融合基因加以证实[27]。在我们回顾的文献中，有17例报告了染色体t（15;17）或者PML-RARα融合基因阳性的特征性表现。此外，有2例出现染色体畸变，表现为iso（17q）和+17，同时15号染色体正常[19],[25]。另外有3例主要通过细胞形态学、免疫表型诊断APL，未报告特征性细胞遗传学或分子生物学特征[17]–[18],[21]。

在我们回顾的上述文献中，男性患者17例，女性患者5例，男女比例3.4∶1，这符合CML中的男性优势[27]。与女性急变者的平均年龄（64岁）相比，男性急变者的平均年龄更低（38岁），约1/3的患者从最初诊断CML到确诊APL急变的时间小于1年，半数以上的患者发生APL急变的时间为1～5年。我们回顾的文献中有2例患者初诊CML时即发生APL急变[7],[21]，其中一例细胞遗传学分析证实染色体异常，5个分裂象表现为46，XY，t（9；22）（q34；q11），4个分裂象表现为47,+8，t（15；17）（q22；q11-22），分子生物学检测PML-RARα融合基因、BCR-ABL融合基因均阳性[7]，遗憾的是其未完善FISH检测。另一例细胞遗传学分析及FISH检测显示t（9；22），未见t（15；17），最终结合形态学表现诊断为CML急变APL，进而确定适当的治疗方案。

本例患者从CML确诊到APL急变的时间仅为20 d。在确诊APL急变后我们回顾性分析其初诊时骨髓标本，发现其初诊时即存在PML-RARα融合基因（5％）。细胞遗传学分析及FISH检测证实存在两群异常克隆细胞系（共20个分裂象），两种融合基因来源于同一细胞系（7个分裂象），另一群异常克隆细胞系仅检测到BCR-ABL融合基因（13个分裂象）。我们考虑初诊时仅BCR-ABL融合基因阳性的异常细胞系为干系，PML-RARα融合基因阳性的异常细胞系为旁系，患者在经过初期的羟基脲治疗后出现骨髓抑制，骨髓抑制恢复后，旁系异常细胞系发展为干系细胞系，早幼粒细胞异常增殖发生APL急变。

诱导分化治疗极大改善了APL患者的生存情况，在我们复习的文献中，仅8例接受诱导分化治疗，其中4例治疗有效（疗效评估达CR或部分缓解）；接受诱导分化治疗的患者中，仅2例接受异基因造血干细胞移植，1例疗效评估达CR，另1例于移植后第118天死亡。鉴于CML急变的APL极其罕见，这类患者是否需要异基因造血干细胞移植仍需进一步研究。
